# Eligibility for subcutaneous implantable cardioverter-defibrillator in patients with left ventricular assist device

**DOI:** 10.1007/s10840-020-00810-1

**Published:** 2020-07-01

**Authors:** Christos Zormpas, Jörg Eiringhaus, Henrike A. K. Hillmann, Stephan Hohmann, Johanna Müller-Leisse, Jan D. Schmitto, Christian Veltmann, David Duncker

**Affiliations:** 1 Hannover Heart Rhythm Center, Department of Cardiology and Angiology, Hannover Medical School, Carl-Neuberg-Str. 1, 30625 Hanover, Germany; 2 Department of Cardiac, Thoracic, Transplant and Vascular Surgery, Hannover Medical School, Hanover, Germany

**Keywords:** ICD, S-ICD, LVAD, Device-device interference, S-ICD screening test

## Abstract

**Purpose:**

The subcutaneous implantable cardioverter-defibrillator (S-ICD) could be a promising alternative to the conventional transvenous ICD in patients with LVAD due to its reduced risk of infection. However, surface ECG is altered following LVAD implantation and, since S-ICD detection is based on surface ECG, S-ICD could be potentially affected. The aim of the present study was to analyze S-ICD eligibility in patients with LVAD.

**Methods:**

Seventy-five patients implanted with an LVAD were included in this prospective single-center study. The ECG-based screening test and the automated screening test were performed in all patients.

**Results:**

Fifty-five (73.3%) patients had either a positive ECG-based or automated screening test. Out of these, 28 (37.3%) patients were found eligible for S-ICD implantation with both screening tests performed. ECG-based screening test was positive in 50 (66.6%) patients; automated screening test was positive in 33 (44.0%) patients. Three ECG-based screening tests could not be evaluated due to artifacts. With the automated screening test, in 9 (12.0%) patients, the test yielded no result.

**Conclusions:**

Patients implanted with an LVAD showed lower S-ICD eligibility rates compared with patients without LVAD. With an S-ICD eligibility rate of maximal 73.3%, S-ICD therapy may be a feasible option in these patients. Nevertheless, S-ICD implantation should be carefully weighed against potential device-device interference. Prospective studies regarding S-ICD eligibility before and after LVAD implantation are required to further elucidate the role of S-ICD therapy in this population.

## Introduction

Implantable cardioverter-defibrillators (ICDs) represent an established therapy to reduce sudden cardiac death in patients with symptomatic heart failure and reduced left ventricular function [1]. Implantation of left ventricular assist devices (LVAD) in patients with end-stage heart failure has led to a significant improvement in survival rates and patient’s quality of life [2, 3]. The implantation was initially meant as bridge to transplantation, though in recent years the procedure is increasingly performed as destination therapy [2]. Since LVAD implantation is performed in advanced heart failure, in most of the patients, an ICD is indicated.

Conventional transvenous ICD systems carry a significant risk for peri-procedural complications, such as pneumothorax, pericardial effusion, hemothorax, and lead dislodgement and also for chronic complications, including endocarditis, thrombosis, and lead failure [4–6]. In order to avoid these complications, subcutaneous ICD (S-ICD) systems have been developed [7]. S-ICD therapy has been shown to be a safe and effective alternative to the transvenous ICD [8].

ICD therapy in LVAD patients can be challenging and several studies have reported serious side effects derived from the co-existence of ICD and LVAD, including lead failure and telemetry failure [9, 10]. Especially, device-device interferences have been reported in patients with LVAD implanted with an S-ICD [11–13].

**Fig. 1:**
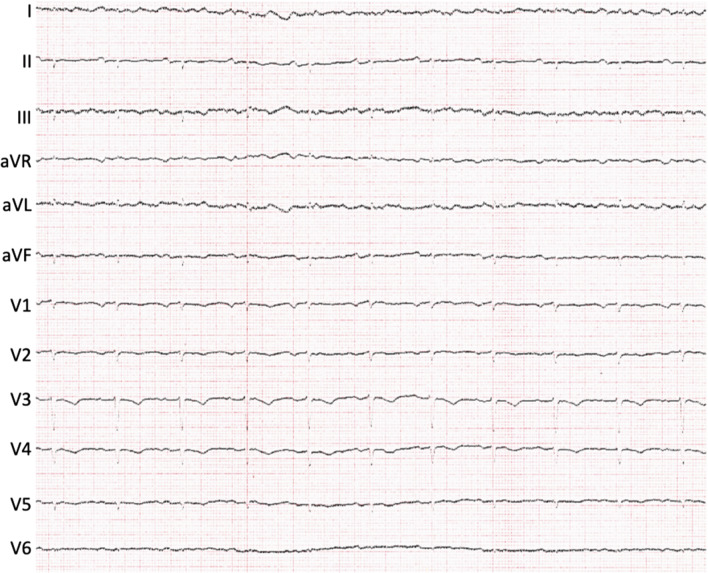
Twelve-lead ECG of a patient with an implanted LVAD (HVAD). Typical ECG characteristics: high-frequency artifacts particularly in leads I, III, as well as V5 and V6 and low QRS amplitude [14]

The advantage of the S-ICD is the avoidance of transvenous intracardiac leads, which could be particularly beneficial in patients with an LVAD. Adequate S-ICD sensing is based on the subcutaneous lead and relies on good discrimination between P, R, and T waves. Electrocardiographic changes may occur after LVAD implantation [14] which consecutively may impact proper sensing of the S-ICD system and thus S-ICD eligibility (Fig. 1). ECG-based S-ICD screening test interpretation can be challenging in patients with LVAD.

The aim of the present study was to evaluate S-ICD eligibility in patients implanted with an LVAD using the available screening methods and to identify parameters affecting S-ICD eligibility in these patients.

## Methods

Consecutive patients implanted with an LVAD at Hannover Medical School presenting for routine follow-up were included in the study in a prospective non-randomized manner. The study complied with the Declaration of Helsinki and was approved by the local ethics committee. All patients gave written informed consent.

Baseline parameters were recorded including body mass index (BMI) and chest circumference. A standard 12-lead ECG was performed in all patients in accordance with international standards [15].

### S-ICD screening procedure

In all patients, the two available S-ICD screening tests were performed to evaluate S-ICD eligibility: (1) an ECG-based S-ICD screening and (2) an automated S-ICD screening test.

**Fig. 2:**
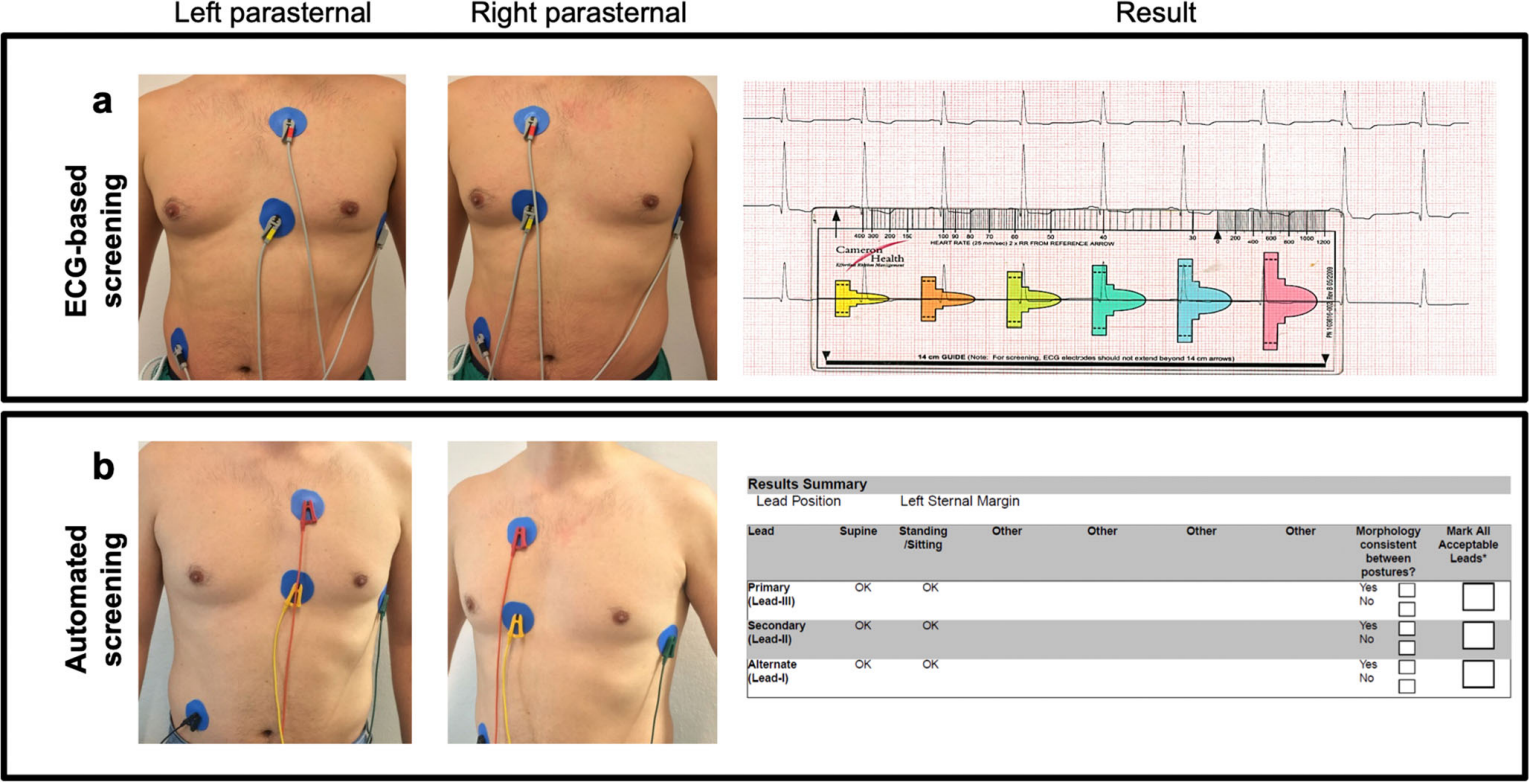
S-ICD screening tests and result. ECG-based screening test (**a**) and automated screening test (**b**) in left parasternal and right parasternal positions

**Fig. 3:**
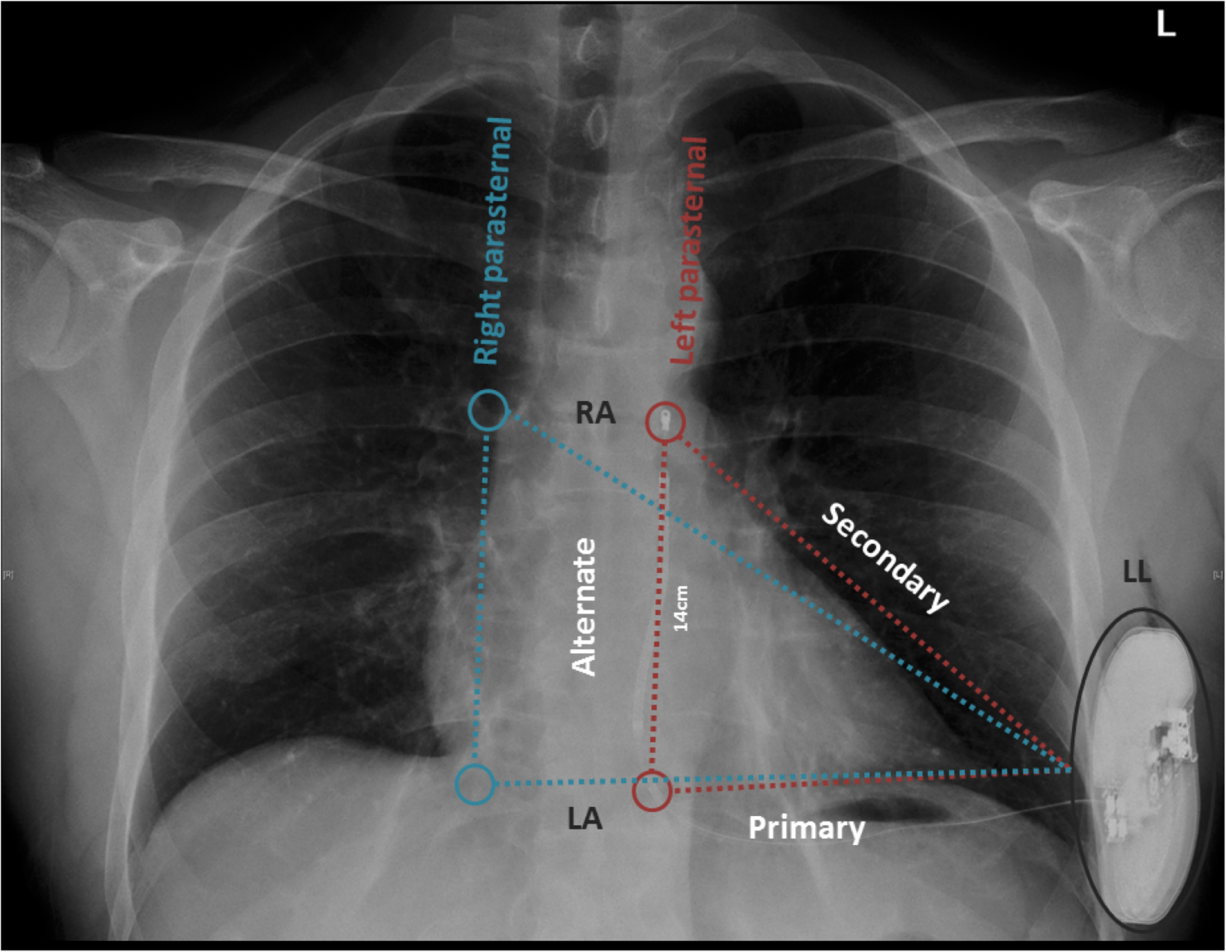
Chest X-ray of a patient with an implanted S-ICD in the left parasternal position. The 3 possible vectors formed from the S-ICD lead and the S-ICD can are depicted. Red colored are the vectors formed in the left parasternal position and blue colored are the vectors formed in the right parasternal position. RA, right arm; LA, left arm; LL, left leg

For the ECG-based screening test, standard ECG limb electrodes (LA, RA, LL) were placed as follows (Fig. 2a): (1) 1 cm lateral to the xiphoid process (LA), left and right parasternal, respectively, (2) 14 cm cranial to the first electrode (RA), and (3) on the left mid-axillary line, 5th or 6th intercostal space (LL). The neutral electrode was placed on the right lower abdominal wall. With this electrode configuration, S-ICD sensing vectors were simulated in the left parasternal and right parasternal position as depicted in Fig. 3. For each patient, screening test was performed in supine and erect positions. For the ECG-based screening, recordings were obtained at gains of 5, 10, and 20 mV at a paper speed of 25 mm/s using an ECG device (MAC 5500, GE Healthcare, Chicago, IL, USA).

Regarding automated screening, vector eligibility was determined from the manufacturer screening template (Latitude Programmer Model 3120, Boston Scientific, Natick, MA, USA). Positioning of the electrodes was performed analog to the ECG-based screening (Fig. 2b).

### S-ICD eligibility

The ECG-based screening test was considered positive if screening template passed in at least one lead in both supine and erect positions at any gain in either left parasternal or right parasternal position. For the automated screening test, S-ICD eligibility was determined automatically if at least one vector was found eligible in both supine and erect position in either left parasternal or right parasternal position.

### Statistical analysis

Categorical variables are presented as numbers and percentages and were compared among subgroups using the chi-square test or the Fisher’s exact test accordingly. Continuous variables are presented as mean ± standard deviation. Differences among continuous variables were compared using an unpaired *t* test. Values of *p* < 0.05 were considered statistically significant. Statistical analysis was conducted using GraphPad PRISM 6 software (GraphPad Software, Inc., CA, USA).

## Results

The study population consisted of 75 patients included between September 2016 and February 2017. Baseline characteristics are shown in Table 1. Inclusion in the study occurred in median 873.5 days after LVAD implantation.

**Table 1:** Baseline patient characteristics. *LVAD*, left ventricular assist device

Parameter	*n* = 75
Age (years)	59.4 ± 9.7
Male (*n*, %)	63 (84.0)
Chest circumference (cm)	106.5 ± 12.0
Body mass index (kg/m^2^)	28.0 ± 5.8
Etiology of cardiomyopathy (*n*, %)	
•Dilated cardiomyopathy	45 (60.0)
•Ischemic cardiomyopathy	26 (34.6)
•Other	4 (5.4)
Prior cardiac surgery (*n*, %)	52 (69.3)
Implanted ICD (*n*, %)	73 (97.3)
Pacemaker dependent (*n*, %)	5 (6.6)
Pacing percentage (*n*, %)	
•< 1%	47 (62.7)
•1–80%	6 (8.0)
•> 80%	22 (29.3)
LVAD type (*n*, %)	
•HVAD	48 (64.0)
•HeartMate III	14 (18.7)
•HeartMate II	11 (14.6)
•HeartAssist5	2 (2.7)
Minimal invasive LVAD operation technique (*n*, %)	34 (45.3)

### Electrocardiographic characteristics

Twelve-lead ECG was available for all patients. Three out of 75 ECGs (4%) showed extensive artifacts from the LVAD and were excluded from the ECG analysis. Twenty-one (29.1%) patients had a paced QRS complex and 51 (70.9%) patients had an intrinsic QRS complex. Table 2 summarizes the 12-lead ECG parameters analyzed.

**Table 2:** Parameters of evaluable 12-lead ECGs (*n* = 72). *LBBB*, left bundle brunch block; *RBBB*, right bundle brunch block; *IVCD*, interventricular conduction delay

Parameter	*n* = 72
Atrial rhythm (*n*, %)	
•Sinus rhythm	48 (66.7)
•Atrial fibrillation	23 (31.9)
•Paced	1 (1.4)
Heart rate (bpm)	76.1 ± 20.5
Cardiac axis (°)	− 29.0 ± 98.3
PR interval (ms) (*n* = 48)	176.5 ± 48.2
QRS duration (ms)	130.5 ± 45.3
QRS morphology (*n*, %)	
•No BBB	25 (34.7)
•LBBB	15 (20.8)
•RBBB	7 (9.8)
•IVCD	4 (5.6)
•Paced	21 (29.1)
QTc interval (ms)	485.0 ± 58.5

### S-ICD eligibility

Overall 900 S-ICD ECGs, namely 2700 potential vectors, were evaluated for eligibility. Using the ECG-based screening test, 3 (4%) tests could not be analyzed due to manifest artifacts from the LVAD and were therefore considered negative. Performance of automated screening in 9 (12%) patients yielded no result despite multiple attempts, and thus the test was considered negative.

Overall, 55 (73.3%) patients had either a positive ECG-based or automated screening test. Twenty-eight (37.3%) patients were found eligible for S-ICD implantation with both screening tests performed, of which 23 (30.7%) had ≥ 2 eligible vectors. Two patients (2.6%) found ineligible in left parasternal position were found eligible in the right parasternal position.

**Fig. 4:**
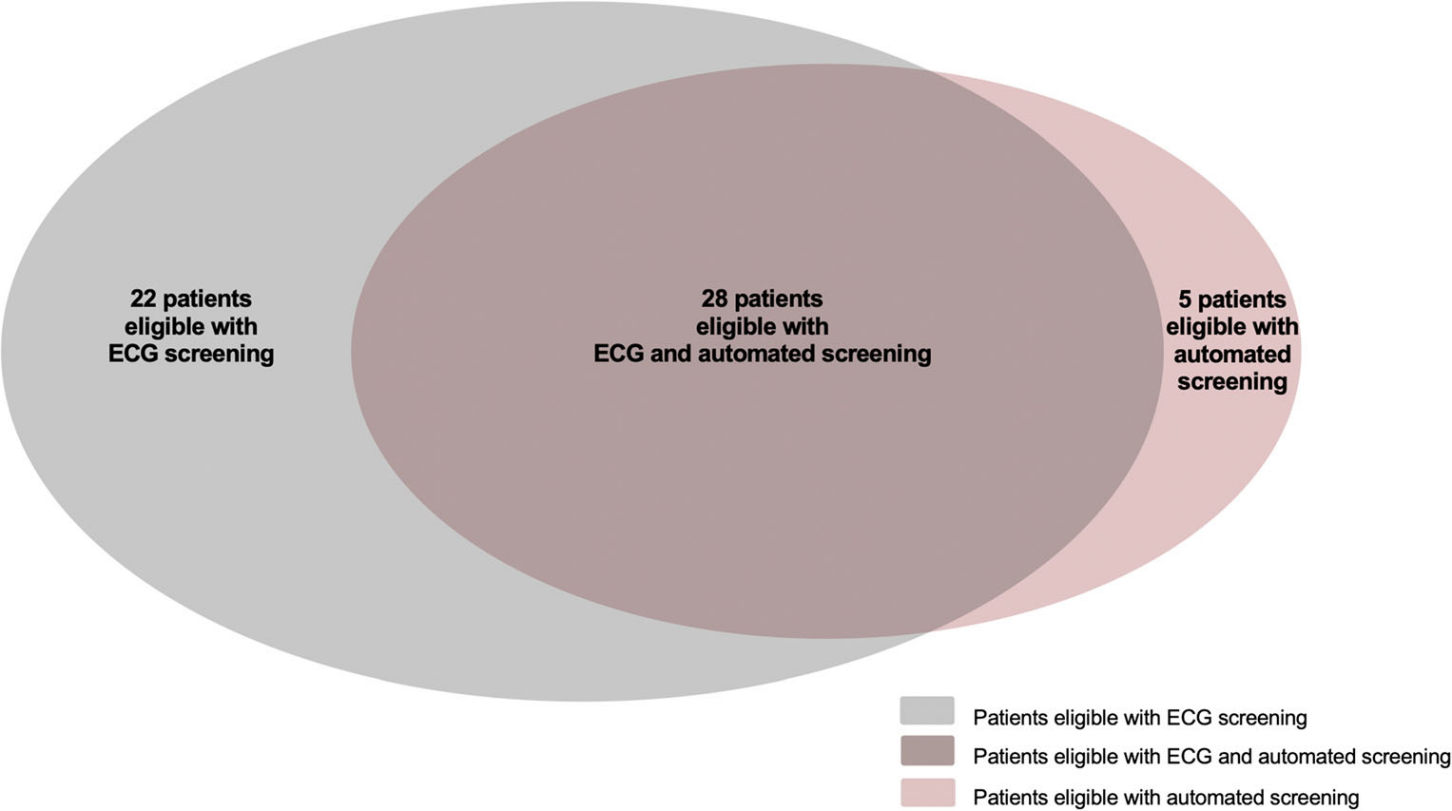
Proportional Venn diagram of 55 patients with an LVAD found eligible for S-ICD implantation according to each screening test performed. The overlapping portion demonstrates the amount of patients with LVAD found eligible for S-ICD implantation with both performed screening methods

Fifty (66.6%) patients were eligible for S-ICD implantation using the ECG-based screening test and 33 (44.0%) patients were eligible with the automated screening test (Fig. 4). Table 3 provides a detailed overview of the S-ICD eligibility results in the different parasternal positions studied and according to the screening test performed.

**Table 3:** Eligible vectors in the different parasternal positions according to the screening method performed in 75 patients

Eligible vectors	ECG-based screening test, left parasternal (*n*, %)	ECG-based screening test, right parasternal (*n*, %)	Automated screening test, left parasternal (*n*, %)	Automated screening test, right parasternal (*n*, %)
0	27 (36.0)	41 (54.7)	45 (60.0)	56 (74.7)
1	25 (33.3)	18 (24.0)	11 (14.7)	7 (9.3)
2	20 (26.7)	14 (18.6)	14 (18.6)	11 (14.7)
3	3 (4.0)	2 (2.7)	5 (6.7)	1 (1.3)

### Reasons for failure of S-ICD screening

With the ECG-based screening test, a total of 2700 S-ICD vector ECGs were analyzed, from which 2168 (80.3%) delivered a negative result. Reasons for failure were a low amplitude of the QRS complex (*n* = 1080, 49.8%), T wave oversensing (*n* = 699, 32.2%), high amplitude of the QRS complex (*n* = 357, 16.5%), oversensing of the following P wave (*n* = 25, 1.2%), and a broad QRS complex not fitting in the QRS-T-wave shell of the screening tool (*n* = 7, 0.3%).

In 9 (12%) patients, the automated screening test yielded no result and was consequently considered negative. Reasons for failure of the automated screening test are not provided by the programming device.

### Factors affecting S-ICD eligibility

In order to evaluate factors which could potentially affect S-ICD eligibility, eligible (*n* = 55) and ineligible (*n* = 20) patients were compared. Table 4 shows an overview of the analyzed parameters comparing both groups. No significant difference was found in any of the analyzed baseline parameters.

**Table 4:** Comparison of baseline parameters between eligible and ineligible patients for S-ICD

Parameter	Ineligible, *n* = 20	Eligible, *n* = 55	*p* value
Age (years)	59.2 ± 7.1	59.5 ± 10.3	0.920
Male (*n*, %)	18 (90.0)	45 (81.8)	0.497
Chest circumference (cm)	107.9 ± 9.7	106.2 ± 13.1	0.533
Body mass index (kg/m^2^)	27.6 ± 5.4	28.2 ± 6.1	0.693
Etiology of cardiomyopathy (*n*, %)			
•Dilated cardiomyopathy	12 (60.0)	33 (60.0)	0.402
•Ischemic cardiomyopathy	7 (35.0)	19 (34.5)	0.705
•Other	1 (5.0)	3 (5.5)	0.997
Prior cardiac surgery (*n*, %)	12 (60.0)	40 (72.7)	0.396
LVAD type (*n*, %)			
•HVAD	14 (70.0)	34 (61.8)	0.299
•HeartMate III	3 (15.0)	11 (20)	0.857
•HeartMate II	3 (15.0)	8 (14.5)	0.966
•HeartAssist5	0	2 (3.7)	0.999
Minimal invasive operation technique (*n*, %)	10 (50.0)	24 (43.6)	0.794
Atrial rhythm (*n*, %)			
•Sinus rhythm	13 (68.4)	32 (60.4)	0.832
•Atrial fibrillation	6 (31.6)	20 (37.7)	0.799
•Paced	0	1 (1.9)	0.999
Heart rate (bpm)	82.1 ± 13.2	75.4 ± 13.7	0.094
Cardiac axis (°)	− 52.7 ± 75.0	− 16.6 ± 107.3	0.097
QRS duration (ms)	134.1 ± 42.9	129.8 ± 35.4	0.705
QRS morphology (*n*, %)			
•No BBB	7 (36.8)	18 (33.9)	0.124
•LBBB	4 (21.1)	11 (20.8)	0.388
•RBBB	0	7 (13.2)	0.388
•IVCD	1 (5.3)	3 (5.7)	0.984
•Paced	7 (36.8)	14 (26.4)	0.388
QTc interval (ms)	471.2 ± 56.5	490.1 ± 59.4	0.427
R:T ratio in lead I	13.7 ± 16.8	9.2 ± 15.9	0.319
R:T ratio in lead II	6.6 ± 11.3	7.7 ± 12.4	0.724
R:T ratio in lead aVF	6.9 ± 11.4	6.5 ± 11.3	0.879

## Discussion

The present study is the first study to assess S-ICD eligibility in a large cohort of patients implanted with LVAD. The main results are as follows:

1. 73.3% of patients with LVAD were eligible for S-ICD implantation either with the ECG-based or the automated screening test.
2. S-ICD eligibility rate of patients with LVAD was higher (66.6%) using the ECG-based screening test in comparison with the automated screening test (44%).

Patients with LVAD may develop device-related (ICD and/or LVAD) infections necessitating extraction of the implanted system [10, 16, 17]. In case of a device infection or lead failure requiring the extraction of the complete ICD system including intracardiac leads, studies have shown a considerable morbidity and mortality [18, 19]. Thus, in these patients, implantation of an S-ICD might be beneficial and could be considered [20]. In patients with LVAD, lack of transvenous access due to abandoned leads and/or recurrent central venous catheters resulting in occlusion of the subclavian veins can pose an important limiting factor in case of lead revision. Thus, S-ICD implantation can overcome several disadvantages of the conventional transvenous ICDs (infection, bleeding, extraction-related complications).

LVAD implantation leads to significant changes of the surface ECG [14, 21]. Moreover, heart failure is a progressive disease which can lead to further ECG alterations [22]. Thus, the S-ICD screening test and consequently S-ICD eligibility could be affected by changes in amplitude and vector of the QRS complex.

The present study shows reduced S-ICD eligibility rates (66.6% with the ECG-based and 44% with the automated screening test) in patients with LVAD compared with previously reported rates in patients with heart failure [23–26]. Nordkamp et al. studied 230 patients with an ICD (primary or secondary prevention) and showed a positive ECG-based screening test in 92.6% of the patients [24]. Similarly, Randles et al. analyzed 196 patients with either primary or secondary preventive indication for ICD therapy and reported S-ICD eligibility rates with the ECG-based screening test of 85.2% [26]. Lower eligibility rates in the present study could be attributed to ECG altering effect of both, the LVAD device itself, the progressive character of end-stage heart failure and the high percentage of patients (29.1%) with a paced QRS complex.

Eligibility for S-ICD in the present study was determined, according to manufacturer’s recommendations, as at least on eligible vector in any of the two screening tests performed. Since S-ICD performance and efficacy in patients with LVAD are poorly known, S-ICD eligibility with both screening tests as well as the ≥ 2 vector rule should be met before considering implantation of an S-ICD in the presence of LVAD. If these two rules are to be applied, the eligibility rates found in the present study are much lower (30.7%). In line with these findings, several case reports of inappropriate ICD therapy in patients with an S-ICD after LVAD implantation have been reported leading to major concerns regarding the safety in case of co-existence of an LVAD and an S-ICD [11, 27, 28]. An additional restricting factor for S-ICD implantation is the lack of antibradycardia therapy, which may be intermittently necessary in some patients. In the present study, 21 (29.1%) patients had a paced QRS complex, although only 5 (6.8%) patients were actually pacemaker dependent. Previous studies have shown slightly reduced S-ICD eligibility rates in patients with CRT or pacemaker devices [29–31]. Giammaria et al. studied S-ICD eligibility in 48 CRT carriers and showed S-ICD eligibility rates of 71% [29]. In the present study, 29.1% of the patients had a paced QRS complex, notwithstanding that the presence of a paced QRS complex did not significantly affect S-ICD eligibility.

Even though only a very small fraction of the patients included were pacemaker dependent (6.8%), it is unclear to which extent intermittent pacing may still be required. Also CRT in non-pacemaker-dependent patients may have a positive effect even in the presence of LVAD, although not adequately studied. The fact that S-ICD lacks also the ability to deliver antitachycardia pacing (ATP) is of great importance for patients with LVAD as they most of the times hemodynamically tolerate VT and thus are conscious in case of high-energy shock delivery.

Clinical performance of S-ICD in patients with LVAD, including proper baseline sensing as well as sensing during arrhythmia, is unknown, particularly when taking into consideration the changing intravascular volume status and electrolyte balance. Major concerns have been also raised regarding defibrillation success after S-ICD pulse generator replacement [32] and the high DFT energy required. Thus, S-ICD eligibility should not be confused with S-ICD efficacy, which was not evaluated in the present study.

In the present study, 12% of the patients with LVAD examined with the automated screening test yielded no result. We hypothesize that this observation was caused by artifacts produced from the LVAD.

Comparison of patients showing eligibility vs. ineligibility did not reveal any significant predictors for S-ICD screening failure. Especially, neither ECG parameters nor the LVAD type or implant technique affected S-ICD eligibility.

In a previous study of 215 patients with LVAD, in which 12-lead ECGs before and after LVAD implantation were analyzed, significant changes of the R:T ratio in leads I, II, and aVF after LVAD implantation were reported [14]. Groh et al. studied 100 patients with an implanted ICD without antibradycardia indication, thus potential S-ICD candidates. Among others, they were able to show the importance of T wave in leads I, II, and aVF of the 12-lead ECG regarding S-ICD eligibility, since T wave inversion in these leads was associated with a 23-fold higher likelihood for S-ICD ineligibility [23]. In the present study, no significant difference in the R:T ratio in leads I, II, and aVF was observed between eligible and ineligible patients.

Further studies comparing S-ICD eligibility before and after LVAD implantation are necessary to elucidate whether these findings are due to the underlying end-stage heart failure or LVAD implantation alone.

### Limitations

The present study is the first to assess S-ICD eligibility in a large cohort of 75 patients with LVAD. It has, however, several limitations. The S-ICD screening tests performed represent a single time point of S-ICD eligibility in median 873 days after LVAD implantation. Thus, it remains unclear to which extent the observed findings are only due to LVAD implantation or progression of the underlying disease. We could not assess eligibility before LVAD implantation and how LVAD implantation affects S-ICD eligibility in each patient. The S-ICD screening test performed in this study did not lead to consecutive S-ICD implantation. Thus, the actual S-ICD failure rates could not be confirmed, especially potential device-device interference in case of an implanted S-ICD, as described in case reports previously [11, 12].

Moreover, in the present study, we observed a quite high percentage (29.1%) of patients with paced QRS complex, which are not primarily considered for S-ICD implantation.

When focusing on each S-ICD screening method alone, it should be emphasized that the ECG-based screening test is an examiner-dependent test, in particular in patients with LVAD due to frequently co-existent artifacts. The automated screening, on the other hand, provides a dichotomic result regarding S-ICD eligibility without any explanation of the reason for failure.

## Conclusion

Patients implanted with an LVAD show an S-ICD eligibility rate of maximal 73.3%. This S-ICD eligibility rate is lower in comparison to patients with heart failure without LVAD. Nevertheless, S-ICD implantation seems to be a feasible alternative for patients with LVAD in selected cases. In patients with end-stage heart failure, in which implantation of an LVAD may become necessary in the near future, implantation of an S-ICD should be carefully weighed against competing risks, since device-device interference can become a significant problem in case of S-ICD implantation. In these individual cases, extensive S-ICD screening testing should be performed to prevent S-ICD failure. Prospective studies are required to further evaluate potential changes in S-ICD eligibility rates after LVAD implantation.

### Availability of data and material

All data are available in the Department of Cardiology and Angiology, Hannover Medical School, Hanover, Germany.
